# Acute myeloid leukemia secondary to acute B lymphoblastic leukemia treated with maintenance therapy in a child: A case report

**DOI:** 10.1002/cnr2.1717

**Published:** 2022-09-26

**Authors:** Xiaoning Wang, Ding Ding, Yalin Liu

**Affiliations:** ^1^ Department of Hematology The First Affiliated Hospital of Xi'an Jiaotong University Xi'an People's Republic of China; ^2^ Department of Pediatric The First Affiliated Hospital of Xi'an Jiaotong University Xi'an People's Republic of China

**Keywords:** acute lymphoblastic leukemia, acute myeloid leukemia, secondary malignancy

## Abstract

**Background:**

Acute lymphoblastic leukemia (ALL) has the highest incidence among childhood hematologic cancers. Exposure to certain cytotoxic therapies for ALL is correlated with a higher risk of secondary malignancies.

**Case:**

We report a rare case of a 6‐year‐old girl being diagnosed with secondary acute myeloid leukemia (AML) during her maintenance phase of treatment for ALL with TEL‐AML1 fusion gene, approximately 17 months after the primary diagnosis.

**Conclusion:**

This case indicates that we should recognize the increased risk of secondary AML for pediatric ALL patients with TEL‐AML1 fusion gene if multiple alkylating drugs and inhibitors for topoisomerase II are included in induction chemotherapy.

## 1 INTRODUCTION

Secondary acute myeloid leukemia (sAML) refers to AML whose occurrence is associated with antecedent hematologic disorders (AHDs) or therapies. Among the existing childhood hematologic cancers, acute lymphoblastic leukemia (ALL) has the highest incidence. With multi‐agent induction, consolidation, and maintenance chemotherapy, children with ALL enjoy a reasonably good survival.^1^, ^2^, ^3^, ^4^ However there are reports of sAML attributable to cytotoxic treatment protocol.^5^, ^6^ sAML usually occurs with a latency period after the treatment of primary malignancies. Amid the first remission of ALL, the cumulative risk of developing sAML was reported to be 1.6% and 4.7% at three‐year and six‐year follow‐up, respectively. In addition, patients having T‐cell and 11q23 chromosomal abnormalities were reported to possess a higher chance of developing sAML.^7^ Therapy related AML (t‐AML) is of two distinct types: type one develops following treatment with alkylating drugs (e.g., cyclophosphamide, melphalan etc.); type two develops following treatment with topoisomerase II inhibitors (e.g., etoposide, doxorubicin etc.). t‐AML secondary to Topoisomerase II inhibitors is generally associated with chromosomal abnormalities, most including KTM2A at 11q23 and RUNX1 at 21q22.^8^

We presented a rare case of a 6‐year‐old girl with sAML while she was receiving maintenance therapy for B‐ALL approximately 17 months after the primary diagnosis. This was the first report that pedatric ALL patients with TEL1‐AML fusion gene had rapid leukemia lineage switching. It indicated that intensive induction and consolidation combined with alkylating drugs and topoisomerase II inhibitors may enhance the risk of secondary malignancies in pediatric TEL1‐AML positive ALL patients with a favorable prognosis. The patient provided written informed consent for the publication of the study.

## 2 CASE

**FIGURE 1:**
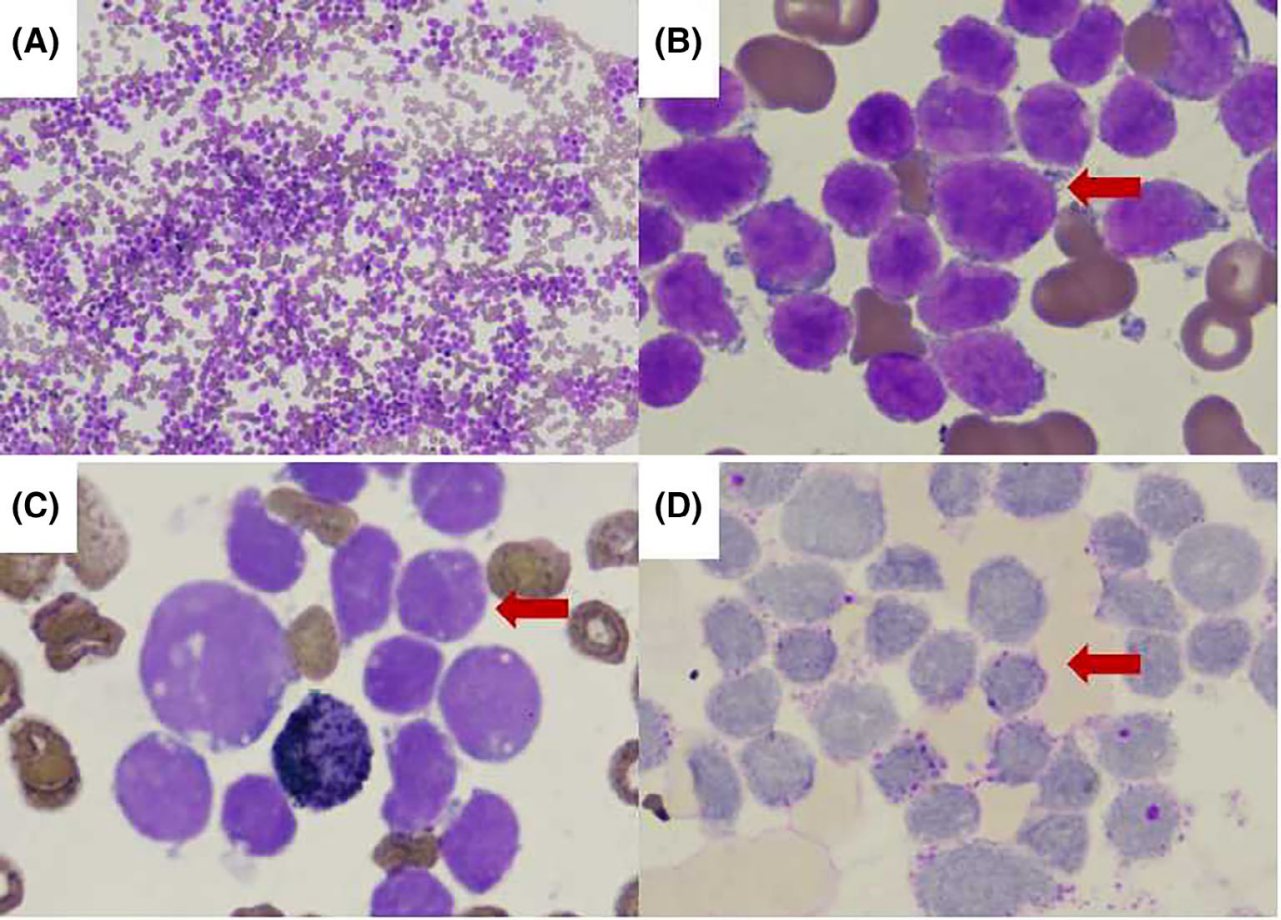
Morphological observations in the bone marrow aspirate smear of the B‐ALL patient. (A) Hematoxylin and eosin staining (H&E) of hypercellula bone marrow smears presenting diffuse infiltration of many large blast cells. (H&E, ×100). (B) Typical lymphoblastic cells with large hyperchromatic and prominent nucleoli (arrow heads, H&E, ×1000). (C) Blast cells are negative for immunohistochemical POX staining (arrow heads, H&E, ×1000). (D) Blast cells are positive for immunohistochemical PAS staining (arrow heads, H&E, ×1000). (E) Chromosome karyotype was 46, XX. (F) Fluorescence in situ hybridization:TEL‐AML1 was positive (arrow heads)

**FIGURE 2:**
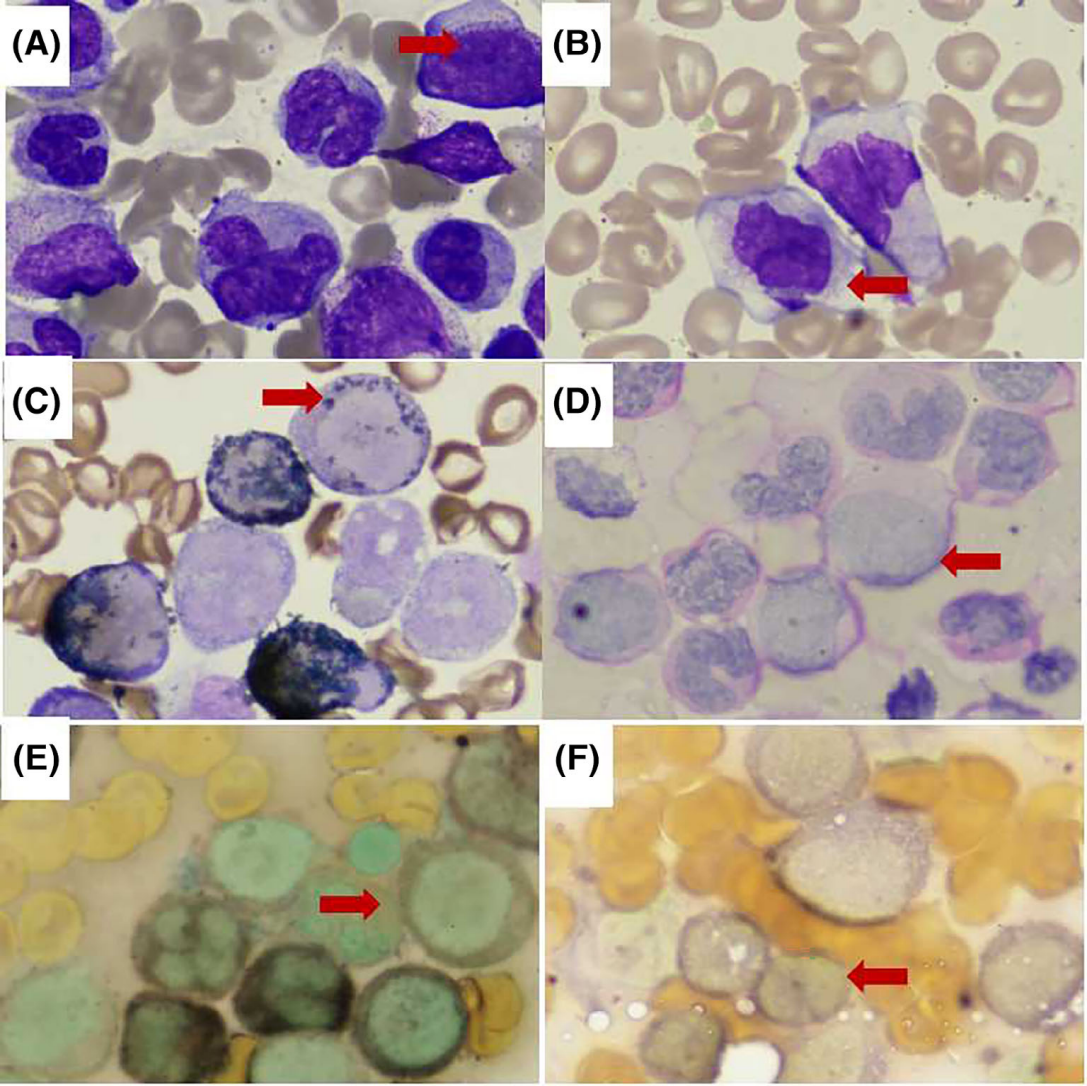
Morphologic findings of secondary AML (A) Typical myeloblastic cells with large hyperchromatic and prominent nucleoli (arrow heads, H&E, ×1000). (B) Blood smear of blast (arrow heads, H&E, ×1000).(C) Blast cells are positive for immunohistochemical POX staining (arrow heads, H&E, ×1000).(D) Blast cells are negative for immunohistochemical PAS staining (arrow heads, H&E, ×1000).(E) Blast cells are negative for immunohistochemical esterase staining (arrow heads, H&E, ×1000). (F) Blast cells are negative for immunohistochemical NAF staining (arrow heads, H&E, ×1000).

**FIGURE 3:**
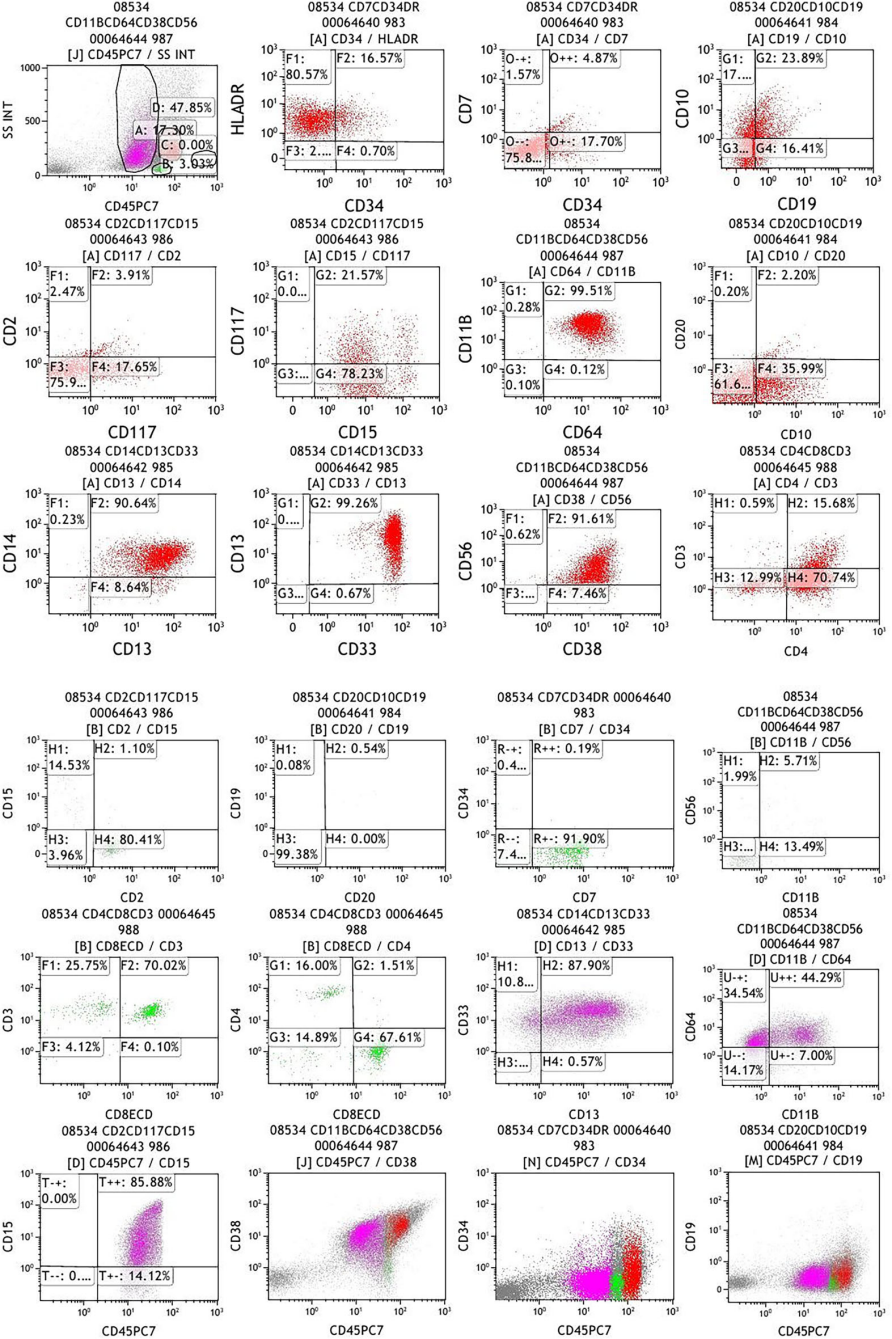
Immunophenotyping of blast cells by flow cytometry. The antibody panels were CD7/CD34/HLA‐DR/CD45/CD20/CD10/CD19/CD45/CD14/CD13/CD33/CD45/CD2/CD117/CD15/CD45/CD11B/CD64/CD56/CD38/CD45/CD4/CD3/CD8/CD45

The patient was admitted to the First Affiliated Hospital of Xi'an Jiaotong University in November 2019 with the chief complain of fever and fatigue for 1 week. A family history of leukemia was denied. There was homogeneous lymphoblastic cells on bone marrow smear. Histochemical staining showed negative POX and positive PAS staining. The chromosome karyotype was 46，XX. TEL‐AML1 was positive detected by fluorescence in situ hybridization (Figure 1). Flow cytometry analysis demonstrated positive expression of CD34, HLA‐DR, CD19, CD22, CD10, CD79a, CD33, CD38, CD13 and negative expression of CD15, CD7, CD117, CD5, CD16, CD4, CD8, CD3, CD20, cMPO, cCD3, CD56, CD14, CD64 on the surface of the blasts. The immnuophenotype of bone marrow supported the diagnose of B‐ALL with myeloid antigens expression. For induction therapy, she was treated with two cycles of quadruple therapy (vincristine, idarubicin, cyclophosphamide, and dexamethasone) and one cycle of triple therapy (cyclophosphamide, cytarabine, and 6‐mercaptopurine). After achieving complete remission, she received 15 cycles of consolidation and maintenance therapies as recommended by the Children's Cancer and Leukemia Group (CCLG)‐2018 protocol. The total dosages of cyclophosphamide and idarubicin were 300 mg/kg and 3.5 mg/kg, respectively. During follow up, the measurable residual disease by flowcytometry and RT‐PCR was constantly negative and the patient only had fatigue. She was admitted to our hospital to receive maintenance therapy in April 2021. On admission, the vital signs were stable. Mild anemia was observed with no tenderness of the sternum. The liver, spleen, and lymph nodes were normal. Laboratory results for the counts of white blood cells (1.46 × 10^9^/L), lymphocytes (0.59 × 10^9^/L), neutrophils (0.81 × 10^9^/L), monocytes (0 × 10^9^/L), eosinophils (0.01 × 10^9^/L), and platelets (112 × 10^9^/L), as well as hemoglobin concentration (84 g/L), were also obtained. The patient exhibited normal serum levels of electrolytes, renal function, and liver function. Bone marrow examination showed increased blasts (Figure 2). Flow cytometry analysis demonstrated positive expression of HLA‐DR, CD14, CD13, CD33, CD15, CD11b, CD38, and CD4, and negative expression of CD7, CD34, CD20, CD19, CD2, CD8, and CD3 on the surface of these blasts (Figure 3). The bone marrow samples were also subjected to G‐banded karyotyping, revealing a 46, XX karyotype. Next‐generation sequencing confirmed 23.46 percents of the *CSMD1* gene mutation (p.Asn1127Lys and p.Pro1995Leu) in bone marrow. And the *CSMD1* gene mutation wasn't detected in oral mucosal epithelium cells. After bone marrow smear, immunophenotyping, and next‐generation sequencing, she was diagnosed with sAML with *CSMD1* gene mutation. The patient underwent induction chemotherapy with EA (etoposide and cytarabine). Subsequently, she underwent a salvage haploidentical hematopoietic stem cell transplant and was then followed up.

## 3 DISCUSSION

ALL has the highest incidence among childhood hematologic cancers. Treatment of ALL includes induction, consolidation, and maintenance therapy, which lasts for no less than 2 years after diagnosis. The cure rate is approximately 90%. There is a risk of developing secondary leukemia following chemotherapy in some patients. The occurrence of sAML can be associated with either AHDs or therapeutic drugs including alkylating drugs and topoisomerase II inhibitors, or radiotherapy. Patients who develop sAML following alkylating drug treatment often have partially or completely deleted chromosome 5 or chromosome 7 and previous myelodysplasia with a 5‐ to 7‐year mean latent period; while patients with topoisomerase II inhibitor‐associated sAML often have chromosomal abnormalities involving chromosome band 11q23 such as *KTM2A* gene,^9^, ^10^, ^11^ and develop leukemia with a relatively short latency period of 1–2 years.

This patient used idarubicin and cyclophosphamide during the treatment protocol, and developed sAML 17 months after primary diagnosis. Idarubicin, a second‐generation anthracycline, has been demonstrated to be effective in treating AML and recurrent ALL.^12^, ^13^, ^14^ But in acute promyelocytic leukemia treated with idarubicin had a higher incidence of secondary cancers compared with non‐chemotherapy regimen.^15^ We speculate that combined with topoisomerase inhibitors and alkylating agents such as idarubicin and cyclophosphamide, which are used in induction therapy for adults, may promote this short‐term disease transformation in children.^16^ For now, clonal hematopoiesis with driver mutations in combination with the features of cytostatic drug metabolism and DNA damage repair are considered a comprehensive marker of an increased risk of t‐AML. So, for treating primary pediatric ALL patients, we may avoid to combine topoisomerase inhibitors with alkylating agents such as idarubicin and cyclophosphamide in induction and consolidation therapy.

It is reported that some cases of childhood leukemia arise prenatally.^17^ Chromosomal translocations, especially those generate the *TEL‐AML1* fusion gene have been detected in peripheral blood at birth ages prior to the onset of leukemia, supporting that leukemogenesis includes multiple steps. This patient had the *TEL‐AML1* fusion gene at the onset of ALL, but consistently negative during maintenance therapy and onset of secondary AML, which suggested that the sAML may have little correlation with *TEL‐AML1* fusion gene.^18^

Individuals affected by Fanconi anemia or aplastic anemia are more likely to develop sAML. According to a recent study, mutations in *GATA2*, *RUNX1*, *TP53*, and *CEBPA* genes were detected in families exhibiting an unexplained high incidence of AML, implying familial susceptibility to AML.^19^, ^20^ Human *CSMD1* is a putative tumor suppressor gene. It is expressed at high levels in the cerebellum, cerebral cortex, white matter of brain, and testis, and low levels in the placenta, thyroid gland, and breast. Loss of alleles, aberrant methylation levels, and mutations of *CSMD1* gene have been implicated in various cancers, including head and neck, breast, lung, liver, colorectal, prostate, and skin cancers, oral squamous cell carcinoma, chronic myeloid leukemia, and recurrent ALL.^20^, ^21^, ^22^, ^23^ In this patient, there was no *CSMD1* gene mutation at the initial diagnosis of ALL and was detected at the onset of sAML, and the *CSMD1* gene mutation in this patient may contribute to the development of sAML.^24^ It was speculated that chemotherapy drugs such as idarubicin and cyclophosphamide may lead to the mutation of *CSMD1* gene which resulted in the development of sAML.

## 4 CONCLUSION

In summary, pediatric sAML rarely occurs while the patient is being treated for primary TEL1‐AML positive ALL with a favorable prognosis. The early‐onset sAML in our patient may be caused by the intensive induction therapy with alkylating agent and topoisomerase II inhibitor she received and the presence of a *CMSD1* mutation. Keeping this possible adverse effect of the treatment regimen in mind can help us recognize the higher risk of secondary malignancies if multiple alkylating agents and topoisomerase II inhibitors are included in induction chemotherapy for pediatric ALL.

## AUTHOR CONTRIBUTIONS

**Xiaoning Wang:** Data curation (equal); writing – original draft (equal). **Ding Ding:** Data curation (equal); resources (equal). **Yalin Liu:** Writing – review and editing (equal).

## FUNDING INFORMATION

This study was supported by the Key Research and Development Program of Shaanxi Province (2020SF‐174, 2020SF‐176, 2021SF‐302 and 2022SF‐13)

## CONFLICT OF INTEREST

The authors have stated explicitly that there are no conflicts of interest in connection with this article.

## ETHICS STATEMENT

Written informed consent was received from the patient, and the study was approved by the ethics committees of the first affiliated hospital of Xi'an Jiaotong Univeristy.

## Data Availability

The data that support the findings of this study are available from the corresponding author upon reasonable request.
